# Emotional Empathy as a Mechanism of Synchronisation in Child-Robot Interaction

**DOI:** 10.3389/fpsyg.2018.01852

**Published:** 2018-10-16

**Authors:** Irini Giannopulu, Kazunori Terada, Tomio Watanabe

**Affiliations:** ^1^Interdisciplinary Centre for the Artificial Mind (iCAM), Faculty of Society & Design, Bond University, Gold Coast, QLD, Australia; ^2^Department of Electrical, Electronic and Computer Engineering, Gifu University, Gifu, Japan; ^3^Department of Systems Engineering, Okayama Prefectural University, Okayama, Japan

**Keywords:** child, emotional empathy, interactor robot, heart rate, synchronisation

## Abstract

Simulating emotional experience, emotional empathy is the fundamental ingredient of interpersonal communication. In the speaker-listener scenario, the speaker is always a child, the listener is a human or a toy robot. Two groups of neurotypical children aged 6 years on average composed the population: one Japanese (*n* = 20) and one French (*n* = 20). Revealing potential similarities in communicative exchanges in both groups when in contact with a human or a toy robot, the results might signify that emotional empathy requires the implication of an automatic identification. In this sense, emotional empathy might be considered a broad idiosyncrasy, a kind of synchronisation, offering the mind a peculiar form of communication. Our findings seem to be consistent with the assumption that children’s brains would be constructed to simulate the feelings of others in order to ensure interpersonal synchronisation.

## Introduction

The term *empathy* derives from the Ancient Greek word “εμπάθεια” (“εν πάθ**o**ς,” i.e., in passion), which has been used to create the German word “*Einfühlung”* that means “feeling into.” Empathy has been defined as a multifaceted process encompassing emotional and cognitive components (Christov-Moore et al., 2014). In this study, we focus mainly on emotional empathy. In a human-human interactive process, emotional empathy leads one to discern the feelings of another and reflects the matching between “self” and the “other’s” feelings (Kanske, 2018). As such, emotional empathy embraces the similar sharing of the positive or negative emotional state of the other person which can be generated by direct interpersonal communication (Lamm et al., 2017).

Emotional empathy has been shown to be a premise of the synchronisation between children and their mothers. When 2 to 3 years of age, children seem enable to anticipate the emotional states of others (Tramacere and Ferrari, 2016). Developmental references suggest that children aged 6 to 7 years old can recognise and respond empathically to positive and negative emotions of their animate or inanimate companions in complex situations (Winnicott, 1971). As children grow into different environments with contrasting conventions, including emotion, it might make sense that they build different ideas about the meaning that is associated with those conventions. Many options have been utilised to explore interpersonal communication. Whereas contextual factors are thought to be crucial for designing better interpersonal interaction, theories eulogise that children are born with an integrated feeling and expression of their emotional selves in their relationship with the others (Frith and Frith, 2003). In human-human interaction, emotional adjustment between companions is based on synchronisation and relationship (Lischke et al., 2018). Therefore, it has been demonstrated that when people interact verbally and non-verbally face to face, they naturally synchronise their reactions (Llobera et al., 2016; Cornejo et al., 2017).

Beyond interpersonal interaction studies, some cross-educational investigations exist on how children interact with robots. Even so, these investigations are not convincing. Some studies have indicated that when robots were used as companions several aspects of human–human communication were directly replicated in human–robot communication (Audrey, 2009). Some others have shown that the way humans communicate with robots is education-dependent (Castellano et al., 2010; Shahid et al., 2014). Based on observations or on self-report questionnaires, most of the above-mentioned studies were conducted more frequently with adults than with children using anthropomorphic or zoomorphic robots (Mitchell and Hamm, 1997). Only marginal attention was committed to the comparison between human-human and human-robot interaction using toy robots (i.e., non-anthropomorphic or non-zoomorphic robots). Moreover, in the aforementioned cross-educational studies, the question of a synchronisation mechanism between companions (human and/or robots) has never been investigated. However, this question is fundamental when we explore human-robot interaction (Giannopulu, 2016a,b, 2018).

In highlighting the importance of synchronisation, our view is congruent with the neuroconstructivist position. According to this position, emotional development, including emotional empathy, arises from dynamic contextual changes in neural structures leading to construal representations across multiple brain regions (Marschal et al., 2010). As such, these representations not only depend on the neural context but also on the physical context (Cacioppo et al., 2014). The analogy between neural activity during experiences has further motivated the interpretation of emotional empathy as a simulation process, associated with a robust biomarker: the mirror neuron system (Rizzolatti and Craighero, 2008). Neuroscientific evidence indicates that there are suggestive parallels between the emotional experience of “self” and “others” (Lamm et al., 2011). Developing areas such as the amygdala, posterior insula, and ventromedial pre-frontal cortex share emotional empathy in children aged 6 to 7 years old even if they show changes in functionality over a lifespan (Decety and Michalska, 2010; Steinbeis et al., 2015; Tramacere and Ferrari, 2016). Subcortical areas (i.e., midbrain areas), develop in association with these other areas (Fan et al., 2011) emphasising the possibility of automatic and unconscious functioning (Giannopulu and Watanabe, 2015, 2018; Giannopulu et al., 2016, 2018; Giannopulu, 2018). Actually, it has been revealed that emotional empathy is directly associated with an autonomic activity mediated by heart rate (Müller and Lindenberger, 2011). Physiological techniques have therefore reported evidence of heart rate synchronisation between adults partners (Levenson and Gottman, 1985) and mother-child dyads (Feldman et al., 2011). Individuals with a high level of emotional empathy showed high heart rates (Stellar et al., 2015; Lischke et al., 2018) and reported hardly any difficulties in recognising and expressing their own emotional feelings (Panayiotou and Constantinou, 2017).

In the context of an international and interdisciplinary project on human-human and human-robot interaction using a verbal and non-verbal communication between two actors, a speaker and a listener, we investigated emotional empathy in two groups of children: French and Japanese. The speaker was always a neurotypical child, and the listener was a human or a toy robot named “Pekoppa” that reacted to speech sounds by nodding (Watanabe, 2011; Giannopulu, 2016a,b; Giannopulu et al., 2016, 2018). Based on a mathematical construction, the toy robot automatically generated nodding motion from speech input which promoted the synchronisation with the speaker (Watanabe, 2011). Specially designed for human-robot intercommunication, this toy robot is a universal listener. The human in the study was the same in France and in Japan, and carried out the same procedure in both countries. The range of research discussed so far lends implicit support to the inference that the use of a universal synchroniser such as the toy robot “Pekoppa” would lead to similarities in communicative exchanges between French and Japanese children. Namely, in each group, children would discern all communicative signs in terms of their understanding of the given empathetic state behind the behaviour of others: human or robot. Accordingly, we have hypothesised that emotional empathy, which is a mechanism of synchronisation, would lead to potential similarities between companions (human-human and human-robots) both in France and in Japan.

## Materials and Methods

### Participants

Two groups of 6-year old children participated in the study. Twenty children (10 boys and 10 girls) composed the “French Group”; twenty children (10 boys and 10 girls) composed the “Japanese Group.” The developmental age of the first group ranged from 6 to 7 years old (mean = 6.3 years; s.d = 4 months). The developmental age of the second group ranged from 6 to 7 years old (mean = 6.4 years; s.d = 2.4 months). The children came from the same class both in Paris and Gifu. As reported by their parents and themselves, none of them had previous experience with robots. All the children were healthy. As reported by their teachers, children attended regular schools and had no learning disorders, neurodevelopmental diseases or cardiac or psychiatric problems. Their academic achievements were standard in their schools. The study was approved by the local ethical committee in Paris (Scientific Committee of Individual’s Protection), France and Gifu (Medical Review Board of Gifu University Graduate School of Medicine) Japan and was in accordance with Helsinki convention 2.0. Anonymity was guaranteed. In both countries, parents gave their informed consent both verbally and in writing for the participation of their children in the study as well as for the analysis of the data; however, they did not allow the authors to send out the raw data. In addition, in Paris and Gifu, each child was asked to give his/her verbal consent before the study began.

### Robot

**FIGURE 1:**
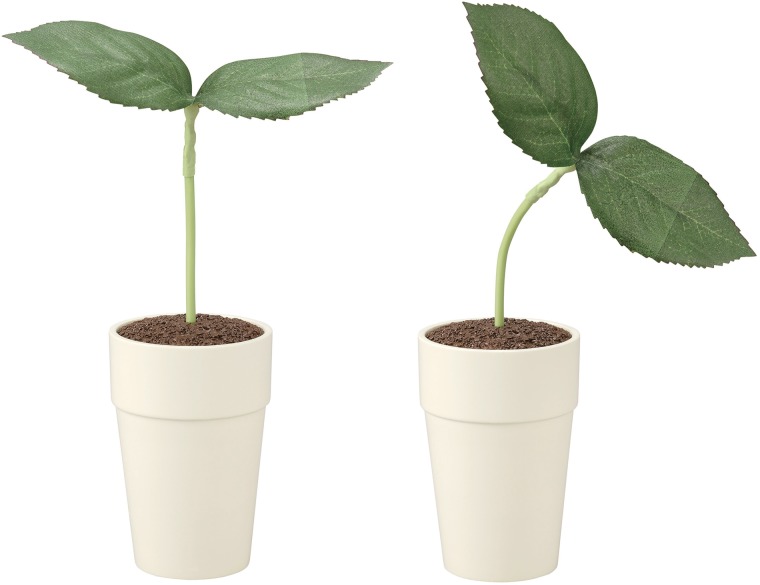
InterActor toy robot (Giannopulu et al., 2016).

An InterActor toy robot, called “Pekoppa,” was used as a listener (Watanabe, 2011). Pekoppa is the simplest expression of Sakura which is a humanoid robot that reacts to speech sounds by only nodding in the same way as humans do. Pekoppa is shaped like a bilobed plant and its leaves and stem make a nodding response based on speech input and supports the sharing of mutual embodiment in communication (see **Figure 1**). It uses a material called BioMetal made of a shape-memory alloy that acts as its driving force.

### Heart Rate Device

A Mio Alpha watch heart-rate monitor was used to record heart rate. It was systematically placed on the left hand of each participant in Paris and Gifu. Mio Alpha measures the heart rate on-line using two green LED and a photo-electric cell. The LEDs are integrated in the back plate of the watch. They project light on the skin, which allows the photo-electric cell to detect the volume of blood flow. The optical sensor displays an accuracy of -01 ± 0.3 bpm. Universal in nature, the device can be used across the lifespan. However, the physiological heart rate limits differ according to the age of individuals. At the age of 6 to 7 years, the heart rate corresponds to 95 bpm (± 30).

### Procedure

**FIGURE 2:**
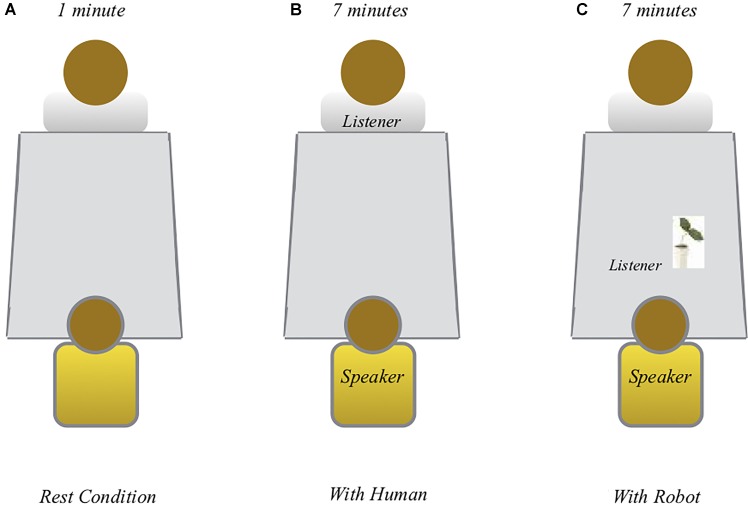
Listener-speaker scenario (Giannopulu et al., 2016).

For both groups, the study took place in a room with which the children were familiar. The room was located in a school context both in Paris and Gifu. We defined three conditions: the first was called “rest condition,” the second was called “with human,” i.e., child-adult, and the third was called “with robot” (i.e., child-robot). The second and third conditions were counterbalanced across the children. The duration of the “rest condition” was 1 min; the second and third conditions each lasted approximately 7 min. The inter-condition interval was approximately 30 s. For each child, the whole experimental session lasted 15 min (see **Figure 2**).

At the beginning of each session, the experimenter presented the robot to the child explaining that the robot nods whenever the child speaks. Then, the experimenter hid the robot. The session was run as follows: during the “rest condition,” the heart rate of each child was measured in silence. At the end of that condition, the child was also asked to estimate her/his own emotional feeling on a scale ranging from 1 (the lowest level) to 5 (the highest level) (Giannopulu and Sagot, 2010; Giannopulu, 2011, 2013; 2016a; 2016b; Giannopulu and Watanabe, 2014; Giannopulu et al., 2016, 2018). Each level corresponded to a specific emotional state depicted by a child’s face as follows: 1st fair, 2nd moderately good, 3rd good, 4th very good, 5th excellent. During the “with human” condition, the child was invited to talk to the experimenter. To that end, the experimenter asked the child “what did you do at school since this morning.” As such, the experimenter initiated discussion and then listened by only nodding to the child. The heart rate of each child was measured meanwhile. During the “with robot” condition, the robot was set to nod; the experimenter gave the robot to the child and invited the child to use it. As previously, the experimenter asked the child to tell the robot “what he or she did at school since this morning.” The robot was the listener, the child was the speaker and the experimenter remained silent and discreet. The heart rate was recorded at the same time once again. The study started around 2.00pm Parisian and Gifu time for all the children. At the end of the session, the child was invited to estimate his/her own emotion on the same above-mentioned scale. More particularly, each child was asked to report his/her own emotional feeling after contact with the robot (Giannopulu and Watanabe, 2015; Giannopulu, 2016a,b, 2018; Giannopulu et al., 2016, 2018).

### Analysis

The heart rate served as the first dependent variable in a 3 (“Rest,” “Human InterActor,” and “Robot InterActor”) × 2 (“French” vs. “Japanese”) mixed-model ANOVA. The emotional feeling reported served as the second dependent variable using the Wilcoxon matched-pairs signed-ranks test. We also performed a statistic of comparisons using the student *t*-test to examine differences in heart rate and chi-square test to analyse the emotional feeling reported. The obtained results were very similar. We present below the results of ANOVA and Wilcoxon matched-pairs signed-ranks test. The data analysis was performed with SPSS Statistics 24.

## Results

First, we present the results for the heart rate of both groups in three conditions: “rest,” “with human,” and “with robot.” We then examine the emotional feeling reported for each group.

**FIGURE 3:**
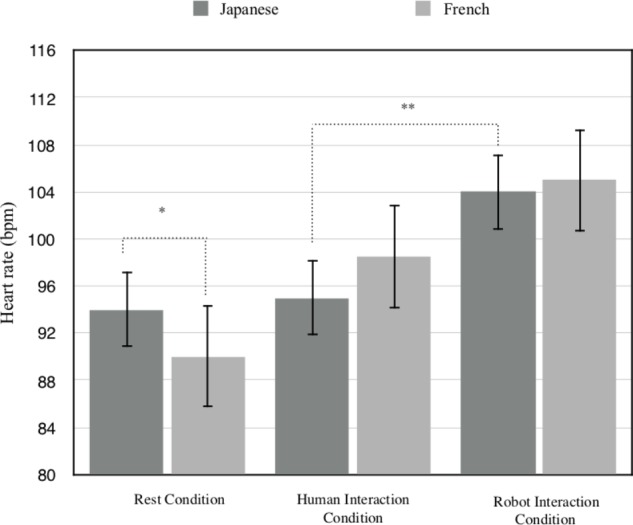
Heart rate comparison between neurotypical Japanese and French children in ‘rest,’ ‘with human,’ and ‘with robot’ condition (^∗^*p* < 0.05; ^∗∗^*p* < 0.01).

As illustrated in **Figure 3**, the mean heart rate of Japanese children was higher (*M* = 94.90, s.d = 7.60) than the mean heart rate of French children (*M* = 89.95, s.d = 5.1) in the “rest condition” (*F*(1,38) = 5.86, *p* < 0.05, $\eta_{p}^{2}$ = 0.1336). Conversely, the mean heart rate of Japanese children (*M* = 95.56, s.d = 7.87), did not differ from the mean heart rate of French children (*M* = 98.2, s.d = 9.39) when the InterActor was the human (*F*(1,38) = 0.93, *p* = 0.3409, $\eta_{p}^{2}$ = 0.0238). In the same vein, the mean heart rate of Japanese children (*M* = 104.06, s.d = 4.08) did not differ from the mean heart rate of French children (*M* = 105, s.d = 6.97) when the InterActor was the robot (*F*(1,38) = 0.22, *p* = 0.6417, $\eta_{p}^{2}$ = 0.0057). Likewise, the mean heart rate in the “rest condition” did not differ from the mean heart rate in the “with human” condition (*F*(1,38) = 0.03, *p* = 0.8633, $\eta_{p}^{2}$ = 0.0007 for the Japanese and *F*(1,38) = 3.67, *p* = 0.0746, $\eta_{p}^{2}$ = 0.0880 for the French children). However, the mean heart rate in the “with robot” condition was higher than the mean heart rate of the “rest condition” for both groups (*F*(1,38) = 17.35, *p* < 0.01, $\eta_{p}^{2}$ = 0.3134 for the Japanese and *F*(1,38) = 23.54, *p* < 0.01, $\eta_{p}^{2}$ = 0.3825 for the French children). Finally, the heart rate of Japanese children was higher when they interacted with the robot than when they interacted with the human (*F*(1,38) = 18.04, *p* < 0.01, $\eta_{p}^{2}$ = 0.3219), but it did not significantly differ for French children (*F*(1,38) = 4.01, *p* = 0.0524, $\eta_{p}^{2}$ = 0.0954).

**FIGURE 4:**
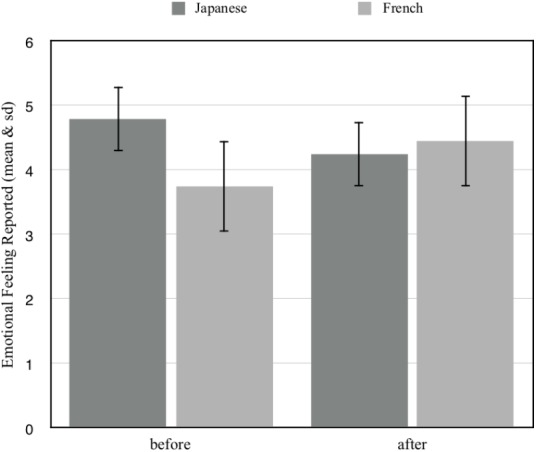
Comparison of Emotional feeling reported ‘before’ and ‘after’ the interaction with the robot in neurotypical Japanese and French children.

**Figure 4** shows that the initial emotional state of French and Japanese children did not differ (Mann–Whitney *U* = 129.5, *p* = 0.0623). In the same vein, the final emotional state of both groups did not differ (Mann–Whitney *U* = 167.5, *p* = 0.3843). The interaction with the InterActor robot did not have any significant effect in the initial emotional state of French and Japanese children (Wilcoxon two-tailed test *p* > 0.05, *T* = 45, *n* = 20 and Wilcoxon two-tailed test *p* > 0.05, *T* = 9, *n* = 20 respectively).

## Discussion and Conclusion

This study dealt with emotional empathy as a mechanism of synchronisation, using the same speaker-listener paradigm and the same human (i.e., experimenter) in two groups of children, one French and one Japanese. It revealed that despite a more important non-verbal emotional empathetic expression in Japanese than French children in the rest condition, a similar mechanism of synchronisation characterised non-verbal communicative exchange when both groups of children interacted with a human or a toy robot. When Japanese children interacted with the robot, their heart rate was higher than when they interacted with the human. Moreover, the initial emotional state reported did not differ for Japanese and French children. The interaction with the toy robot did not affect the final emotional state for either groups of children.

Our results are consistent with recent findings that reported significant interdependence between emotional empathy and synchronisation in adults (Levenson and Gottman, 1985; Stellar et al., 2015; Lischke et al., 2018) and in mother-child dyads (Feldman et al., 2011). But these findings also concur with our hypothesis that emotional empathy which is a mechanism of synchronisation, leads to potential similarities between two different groups of children: French and Japanese. This appears to be reflected in the adjustment of a given automatic physiological state: the heart rate. Physiologically speaking, the heart rate is automatically controlled by both the sympathetic nervous system (SNS) and the parasympathetic nervous (PNS) system of the autonomic nervous system (ANS) and provides a measure of autonomic functioning (i.e., unconscious functioning) (Porges, 2007). The PNS is activated during rest in order to maintain homeostasis; the SNS is activated during periods of perceived change by increasing, the heart rate and mobilising emotional functioning (Suurland et al., 2016). Both physiological systems act in complementary ways to respond and adapt to internal and external changes; namely, both systems are based on synchronisation. Note that the SNS is controlled by the spinal cord, the PNS is controlled by the spinal cord and the brain. Ostensibly, in the rest condition, the heart rate of Japanese children was higher than the heart rate of French children. This automatic activity would provide support for the engagement of the children and would indicate a given emotional state. When Japanese children were in contact with the human or the toy robot, their heart rate was similar to the heart rate of French children.

Contrary to data claiming that autonomic functions in the cardiovascular system largely depend on genetic factors (Tanaka et al., 1994), the present study indicates that these functions seem more likely to rely upon inter communication that is possible via a human or a toy robot in our situation. Essentially, both groups of children expressed potentially similar profiles of heart rate in all conditions, with the exception of a higher heart rate in interaction with the robot than in interaction with the human for the Japanese children. A similar profile was observed for the French children even if the data are not statistically significant. Note that the heart rate of Japanese children was very similar during the “rest” and the “human” conditions where the human was the main passive or active “actor.” Note as well, that the human, and the toy robot, were the same in France and in Japan. In both groups, the heart rate was quasi-identical when both groups of children interacted with the robot.

The sharing and transformation of children’s emotional states would emanate from their understanding of the emotional experience that typifies the other (Giannopulu et al., 2016, 2018; Giannopulu, 2018). Based on interpersonal synchronisation, this is the essence of the speaker-listener condition (Tatsukawa et al., 2016). In this context, both interlocutors are performing a scenario of communication trying various verbal and non-verbal emotional reactions. Verbal reaction necessitates the elaboration of coherent sentences; non-verbal reaction takes the form of head nods and/or various kinds of facial expressions (Giannopulu, 2016a,b, 2018; Giannopulu et al., 2016, 2018; Giannopulu and Watanabe, 2018). Intimately connected with the state of the speaker, these responses signify that everything (or a part) is being integrated (Clark, 1996; Bavelas et al., 2002). Successful communication requires that both speaker and listener accurately interpret (via verbal and non-verbal emotional processes) the meaning of each other’s emotional statement. It seems that the expressive non-verbal emotional nature of a human action (i.e., the heart rate associated with the autonomic nervous system) depicts an indication of the emotional statement of the other at least. This might be considered as an emotional resonance process, or a kind of unconscious synchronisation mechanism that seems to be similar between Japanese and French children. As the emotional state reported is analogous in both populations, it seems that verbal emotional expression would be also associated with this mechanism. This latter result would be consistent with theories in which basic emotional concepts as such “want,” “feel,” or “sense” are common in all educational contexts (Wierzbicka, 1992).

Given the current findings, it seems that both in French and Japanese populations emotional empathy would require the implication of an automatic unconscious identification without any intermediary cognitive empathy (Gallese, 2003; Asada, 2014). Such identification is active with humans and with toy robots. Recent data are in accordance: emotionally empathic children exhibit unconscious non-verbal expressions (Giannopulu and Watanabe, 2018; Giannopulu et al., 2018). Neuroimaging evidence also supports such a process suggesting that the mirror neuron system is involved not only in the intersubjectivity of actions but also in the emotional empathy that allows one to feel related to others (i.e., intersubjective synchronisation) (Carr et al., 2003). Such a neural mechanism authorises one, in essence, to understand the feelings of others as well as to express his or her own feelings (Gallese, 2003). A shared representation of emotional empathetic state underlies the process. In this sense, emotional empathy would be considered a broad unconscious idiosyncrasy offering the mind a specific form of communication: a way of automatic simulation of emotional experiences that is analogous between French and Japanese children. Therefore, our findings are in accordance with the assumption that children’s brains simulate other’s feeling at an unconscious level. This might be valuable for both groups of neurotypical children analysed in our study. Given the above, emotional empathy might be considered as a synchronisation mechanism that supports human-human and human-robot interactions and predicts future emotional behaviours.

## Limitations

One main limitation of our study is the absence of ecological validity. Even if our experimental approach is indispensable to establish valuable relationships between emotional empthy, synchronisation and heart rate, we would suggest that future studies should explore such relationships in more naturalistic contexts. Another limitation of the study is the lack of cortical activity and its relationship with the peripheral activity during the synchronisation process. A possibility for future research could be to investigate whether brain central activity is related to peripheral activity when synchronisation operates in human-human interaction and in child-robot interaction. Finally, we would agree that in our study we did not include a clinical population. Future studies should examine one clinical group (ASD or head injury) at least in comparison with a typical one.

## Author Contributions

IG developed the method, performed the experiment, collected the data, analysed and prepared the manuscript. TW is the creator of the robot. IG, KT, and TW discussed the paper.

## Conflict of Interest Statement

The authors declare that the research was conducted in the absence of any commercial or financial relationships that could be construed as a potential conflict of interest.The reviewer EF and handling Editor declared their shared affiliation.
